# Ambulance clinical placements – A pilot study of students' experience

**DOI:** 10.1186/1472-6920-8-19

**Published:** 2008-04-10

**Authors:** Malcolm J Boyle, Brett Williams, Jennifer Cooper, Bridget Adams, Kassie Alford

**Affiliations:** 1 Monash University, Department of Community Emergency Health and Paramedic Practice, P.O. Box 527, Frankston 3199, Victoria, Australia

## Abstract

**Background:**

Undergraduate paramedic students undertake clinical placements in a variety of locations. These placements are considered an essential element for paramedic pre-employment education. However, anecdotal evidence suggests some students have not had positive experiences on their emergency ambulance placements. The objective of this study was to identify the type of experiences had by students during ambulance clinical placements and to provide feedback to the ambulance services.

**Methods:**

In this pilot study we employed a cross-sectional study methodology, using a convenience sample of undergraduate paramedic students available in semester one of 2007 to ascertain the students' views on their reception by on-road paramedics and their overall experience on emergency ambulance clinical placements. Ethics approval was granted.

**Results:**

There were 77 students who participated in the survey, 64% were females, with 92% of students < 25 years of age and 55% < 65 Kg in weight. There was a statistically significant difference in average height between the genders (Male 179 cm vs Female 168 cm, p < 0.001). Clinical instructors were available to 44% of students with 30% of students excluded from patient management. Thirty percent of students felt there was a lot of unproductive down time during the placement. Paramedics remarked to 40% of students that they doubted their ability to perform the physical role of a paramedic, of this group 36% were advised this more than once.

**Conclusion:**

This study demonstrates that for a small group of students, emergency ambulance clinical placements were not a positive experience clinically or educationally. Some qualified paramedics doubt if a number of female students can perform the physical role of a paramedic.

## Background

Current Australian undergraduate paramedic programs integrate theoretical teaching with clinical placement education in a variety of clinical settings. These settings include non-emergency ambulance, emergency ambulance, hospital wards and departments, community health centres, and rehabilitation centres for variable periods of time. This linkage between theory and practice (praxis) is critical to undergraduate paramedic programs as many have limited scope or capacity for clinical education periods. This potential reduction is largely due to timetabling constraints, health care organisations not able to meet the demands of clinical placements [1], often in direct competition with other allied health care professions [2]. Added to this, reduced exposure to critical ill patients [3] and dilution of advanced clinical skills is common amongst practicing paramedics [1], with the short time allocated for clinical experience, this learning period is vital to students' education.

The Bachelor of Emergency Health (BEH) is a full-time, on-campus degree offered at Monash University. Over the three years of the degree, students are provided with 22 weeks of clinical placements, with 17 weeks allocated to emergency ambulance placements. Anecdotal evidence however, suggests some students have not had positive experiences on their emergency ambulance placements.

The teaching units throughout the BEH use a student-centred approach, mainly case-based learning (CBL). The clinical teaching units that incorporate clinical placements build upon CBL and active learning and utilise educational theories of constructivism [4,5], social constructivism [6] and experiential learning [7,8]. These theories adequately support the responsibilities students have to meet whilst learning theory and applying this in a practical sense during classroom study and real-life exposure to patients whilst on clinical placement.

Comparative studies in this area are few with only one published study located that addressed Australian undergraduate paramedic clinical placements [9]. This paper identified that many learning opportunities are wasted during clinical placements for a variety of reasons, with many students feeling dissatisfied with their learning experiences during the clinical placement time. Moreover, learning difficulties, issues and uncertainties over the most appropriate model for clinical placements has been described in many allied health professions, particularly nursing [10-25,9].

Student views about ambulance clinical placements has the capacity to shape and inform how formal and informal learning takes place while on clinical placements and may also be used in the review of the BEH course as part of the quality audit review framework. Feedback to the ambulance services is also part of the ongoing BEH course review process, and seen as particularly important as pre-employment paramedic education is still a relatively new discipline in the tertiary sector in Victoria and Australia.

The objective of this study was to identify the type of experiences had by students during emergency ambulance clinical placements and to provide feedback to the ambulance services.

## Methods

In this pilot study we employed a cross-sectional methodology using a paper-based form to ascertain the students' views on their reception by on-road paramedics and their overall experience on emergency ambulance clinical placements.

The setting for this study was the undergraduate paramedic course conducted at Monash University. We surveyed a convenience sample of undergraduate BEH students in their first, second, and third year of the course who were available to us following their last lecture in week eleven of semester one of 2007.

Paramedic students undertake clinical placements in both Victorian state ambulance services. Victoria is a south eastern state of Australia and covers approximately 227,590 square kilometres with a population of approximately 5.1 million people [26]. The Metropolitan Ambulance Service (MAS) provides the ambulance service for the greater Melbourne metropolitan area which covers roughly 7,694 square kilometres and a population of some 3.7 million people. Rural Ambulance Victoria (RAV) services the remaining 1.4 million people covering roughly 219,896 square kilometres of Victoria.

The de-identified questionnaire contained 12 questions, some with multiple parts. The first six questions sought information about which year of the BEH course the student was enrolled, with the remaining questions seeking demographic and personal information about the student, e.g. gender, age group, height, and physical build. A Likert Scale was used to elicit the responses to a multipart question about the students' experience whilst on the clinical placement. Two open-ended questions sought a narrative response about positive and negative experiences during the clinical placement. The final two questions asked the student about comments directed toward them about their physical ability to undertake the role of a paramedic by current practicing paramedics. The height, physical build, and question about physical ability were added following feedback from students after previous clinical placements. Several slightly built female students reported they were advised by paramedics they could not undertake the physical aspects of the job, even though they had passed an ambulance service physical capacity assessment prior to the placement. The remaining questions were from an internal questionnaire given to all students following their ambulance clinical placement which is used to evaluate the benefits and effectiveness of the placements.

Descriptive data analysis and comparisons between groups was undertaken using SPSS (Statistical Package for the Social Sciences Version 14.0, SPSS Inc., Chicago, Illinios, U.S.A.). Descriptive statistics were used to summarise the data and a *t *test was used to compare the difference between genders. Narrative responses were collated into general themes with significant comments reported. The results are considered statistically significance if the P value is < 0.05, all confidence intervals (CI) are 95%.

Ethics approval for the project was obtained from the Monash University Standing Committee for Ethics in Research on Humans.

## Results

There were 77 students who participated in the survey, 44% were in third year, 42% in first year and the remainder in second year. The response rate for first years was 100%, 15% for second years and 76% for third years. Females made up 64% of the respondents, this is in line with the gender distribution for each of the years. Ninety two percent of all respondents were less than 25 years of age. Fifty five percent of the respondents were slightly built, weighing less than 65 Kg, with the majority of respondents, 82%, weighing less than 80 Kg.

The average height of students was 172.3 cm (95%CI 170.3 cm to 174.2 cm), median height was 171 cm, range 152 cm to 196 cm. The average height of male students was 179.1 cm (95%CI 176.4 cm to 181.8 cm), median height was 179 cm, range 168 cm to 196 cm. The average height of female students was 168.3 cm (95%CI 166.4 cm to 170.3 cm), median height was 168 cm, range 152 cm to 183 cm. There was a statistically significant difference in average height between the genders (Male 179 cm vs Female 168 cm, p < 0.001).

The majority of students, 55%, were not made welcome at the ambulance station upon their arrival, however, most students, 93%, stated their ambulance clinical placement was a positive experience. This can be highlighted by some of the positive experience comments: "*feel like part of a team*," "*paramedics were willing to assist in further training and understanding of equipment*," "*I learnt so much and gained an understanding of what it was like to be a paramedic*," and "*crews included me as part of the crew*." A greater number of students, 57%, also highlighted that at least one crew member treated them with distain. These negative experiences can be further explained by the negative experience comments: "*sexist attitudes*," "*being told that my only job was to carry bags*," "*know nothing but not offered any opportunities to practice skills*," "*being ignored*," and "*being told my ALS skills were a play on words*."

The first year students had more positive experiences compared to second and third year students, probably due to their lack of knowledge and skills set and less desire to put their limited "knowledge and skills" into practice. For first year BEH students it was there first ambulance placement and hence they were not expected to do much "hands on" patient care.

There were 84% of respondents who felt that scenarios during the undergraduate course assisted them in the progression to real time patient care. Eighty eight percent of the students obtained some form of "hands on" practical experience, during down time or on the road, whilst on the clinical placement. However, 31% of students were excluded from "hands on" patient management. Eighteen percent of students were left at the ambulance station or had to swap to another crew due to doubling up of the "third person" or a clinical support officer was at the station to work with the crew the student was assigned to.

Clinical instructors (paramedic educators) were only available to 44% of students during their clinical placement time, even though they were advised they would be allocated to one for the duration of the placement. Sixty nine percent of students felt there was a lot of unproductive down time during the placement. Thirty seven percent of students were not given the opportunity to undertake clinical scenarios or practice skills during downtime.

Even though we did not ask students to specifically differentiate between their metropolitan and rural clinical placement, students' comments about their metropolitan experience were less favorable than for their rural experience.

On road paramedics remarked to 40% of students that they doubted their ability to perform the physical role of a paramedic, 82% of these students were females. Of this group 36% were advised this more than once and 87% of this cohort were females.

## Discussion

This study has demonstrated that the Monash University undergraduate paramedic program consists mainly of females who are slightly built and shorter than their male counterparts. Even though most paramedic students stated their ambulance clinical placement was a positive experience a small number did not have a positive experience clinically or educationally. A significant percentage of students were advised by a practicing paramedic that they wouldn't be able to perform the physical role of a paramedic.

Interestingly, the majority of students still had high levels of satisfaction despite not being made to feel welcome at the ambulance station, with over half of the students feeling poorly treated by some paramedic staff. Similar obstacles and learning anxieties were also addressed in an experiential learning study by Ham and O'Rourke where nursing students felt anxious when treated harshly by clinical educators, particularly when asked questions they felt they could not answer [27]. Negative student experiences have not only unconstructive effects on learning but also have an effect on where students seek future employment [28,29]. Better preparation for the clinical instructors should be considered by ambulance service administration, this is particularly important as students are known to be anxious about their placements and exposure to their potential employment organisation [30,31]. Preparing instructors or facilitators is supported in other studies by Grant and McKenna, and Reid-Searl and Dwyer [15,32]. A clinical instructor (CI) is a paramedic currently working for an ambulance service (Emergency Medical Service) who has undertaken a recognised short course in workplace training. The CI normally supervises and directs new graduate paramedics during their graduate year program. The CI may also supervise the undergraduate paramedic student during their clinical placement.

An important element of the BEH degree is educational praxis, particularly real-life patient exposure. Without clinical placements, educational quality could be potentially reduced as students would have to rely on artificial patient simulations for the linkage between theory and practice [33]. Real-life exposure and active learning provides the student with a richer source of learning and experience. An excellent example of active learning during clinical placement education was undertaken by Parker and Emanuel that allowed speech and language therapist students to develop their own personal learning approaches, supporting the notion of self-directed learning during clinical placement education [34].

The clinical placement period in this study employed the theory of experiential learning where the student must self-motivate, initiate and reflect on the learning activity previously undertaken [7,8]. Such learning activities might include: assessing patient injuries, manoeuvring stretchers, assessing blood pressure and pulse rates, and developing communication skills in difficult situations. A vital component of the experiential learning process is critical reflection. The importance of critical reflection is underscored with classroom theory being provided to students on models of critical reflection who are also expected to complete a critical reflection journal whilst on clinical placements. In other words, our aim is for students to reflect on each learning experience they undertake. Without this process they cannot associate metacognitive appreciation and learning a way to learn. Similar findings were also found in the qualitative study by O'Donovan who found that students were not provided with adequate time to reflect whilst on placements [22]. Other nursing studies have also found comparable results [35,36].

Clearly, missed learning opportunities have occurred, which are unfortunately supported by findings in a similar paramedic study by Waxman and Williams [9]. The authors in the study reported learning constraints during clinical placements such as, students being too young, not having enough life experience and the CIs not appreciating or understanding the differences university-trained students now bring to the prehospital system. Other nursing studies have also found that "learning wastage" and being unsupported through a lack of educator support occurred for students whilst on clinical placements [11,37]. We concede many variables are involved in this, however, we would argue that if paramedic staff are unwilling to offer adequate learning support to undergraduate students then the first process – 'doing a skill' is lost. Also, reflection by the student could be significantly deeper and richer if paramedics were willing to offer extra 'knowledge-assistance' by way of clinical scenarios and informal discourse during downtime. Not only would this be productive, but also allow the student to synthesise and elucidate information in an environment that is 'learning-friendly' and supportive of experiential learning. Further research is required on this matter. In particular, why is there a lack of interest from some paramedic educators? Are these learning obstacles fixable? Should clinical placement learning objectives be clearer for both student and paramedic educators?

Positive narrative statements demonstrate that the majority of ambulance crews welcomed the student into the station environment, provided skill sessions during downtime, and involved the student in aspects of the patient management they were able to undertake, given their level of knowledge. Overall the placement was a positive experience for the student where they gained an impression of the paramedic role, especially the team work aspect, and where able to put their knowledge and skills into practice. Negative narrative statements demonstrate that some students were not made welcome at the ambulance station, were ignored, made to feel like a burden on the crew, were not provided any guided skills instruction during downtime, and were not permitted to have any interaction with a patient. This may give the student a distorted view of the profession with experiences like this whilst on the placemen. It certainly does not assist in the process of putting knowledge into practice.

The perception of some on-road paramedics that female student paramedics who are young and slightly built will find the physical aspects of the job difficult is baseless but also misinformed. All undergraduate paramedic students must undergo the same ambulance service physical capacity test as full-time paramedics prior to commencing clinical placements.

We are unable to adequately explain why the second year response rate was so low when compared to the other year response rates.

Following this pilot study a more extensive study of undergraduate paramedic student responses to ambulance and hospital clinical placements will be undertaken using undergraduate paramedic students studying at other universities around Australia to ascertain whether the findings from this pilot study are widespread. Whilst these are pilot results, they represent an important set of data in an emerging health care discipline that has identified a range of learning obstacles in clinical placement education. This will provide both university and ambulance administrators with a clearer educational landscape of clinical placement education and the opportunity to develop potential strategies and solutions.

This study is potentially limited by the small number surveyed and hence the presented results may not be a true representation of all student experiences. As we surveyed students across all three years of the course those with more emergency ambulance placement time may have skewed the results. This study only looked at emergency ambulance clinical placements and did not consider all clinical placements in other locations, thereby potentially biasing the result.

## Conclusion

This study demonstrates that even though most students had a positive experience on their emergency ambulance clinical placement a small group of students did not have a positive experience clinically or educationally. Experiential learning suffers considerably when students are not permitted to actively participate with patient care, something that requires further discussion with the ambulance services. Some qualified paramedics doubt if a number of predominately female students can perform the physical role of a paramedic.

## Competing interests

The author(s) declare that they have no competing interests.

## Authors' contributions

JC, BA and KA conceived the study, developed the questionnaire, compiled the data file, and participated in writing the manuscript. MJB assisted in developing the questionnaire, undertook the data analysis, and participated in writing the manuscript. BW participated in writing the manuscript.

## Pre-publication history

The pre-publication history for this paper can be accessed here:


